# How to behave with paediatric myocarditis: imaging methods and clinical considerations

**DOI:** 10.1093/ehjimp/qyaf025

**Published:** 2025-04-04

**Authors:** Rosalba De Sarro, Nunzia Borrelli, Giulia Pelaia, Alessia Mendicino, Sara Moscatelli, Isabella Leo, Giulia La Vecchia, Giuseppe Mazza, Lucy Castaldo, Antonio Strangio, Martina Avesani, Salvatore De Rosa, Daniele Torella, Giovanni Di Salvo, Jolanda Sabatino

**Affiliations:** Division of Cardiology, Magna Graecia University of Catanzaro, Viale Europa, 88100 Catanzaro, Italy; Adult Congenital Heart Disease Unit, AO dei Colli, Monaldi Hospital, Naples, Italy; Paediatric Unit, Department of Experimental and Clinical Medicine, Magna Graecia University, Viale Europa, 88100 Catanzaro, Italy; Paediatric Unit, Department of Experimental and Clinical Medicine, Magna Graecia University, Viale Europa, 88100 Catanzaro, Italy; Centre for Inherited Cardiovascular Disease, Great Ormond Street Hospital, London, UK; Institute of Cardiovascular Sciences, University College London, London, UK; Division of Cardiology, Magna Graecia University of Catanzaro, Viale Europa, 88100 Catanzaro, Italy; Department of Cardiovascular and Pulmonary Sciences, Catholic University of the Sacred Heart, Largo Francesco Vito, 1, Rome, Italy; Center of Excellence of Cardiovascular Sciences, Ospedale Isola Tiberina—Gemelli Isola, Rome, Italy; Division of Cardiology, Magna Graecia University of Catanzaro, Viale Europa, 88100 Catanzaro, Italy; Paediatric Unit, Department of Experimental and Clinical Medicine, Magna Graecia University, Viale Europa, 88100 Catanzaro, Italy; Division of Cardiology, Magna Graecia University of Catanzaro, Viale Europa, 88100 Catanzaro, Italy; Paediatric Cardiology Unit, Department of Woman’s and Child’s Health, University Hospital of Padua, Padua, Italy; Division of Cardiology, Magna Graecia University of Catanzaro, Viale Europa, 88100 Catanzaro, Italy; Division of Cardiology, Magna Graecia University of Catanzaro, Viale Europa, 88100 Catanzaro, Italy; Paediatric Cardiology Unit, Department of Woman’s and Child’s Health, University Hospital of Padua, Padua, Italy; Division of Cardiology, Magna Graecia University of Catanzaro, Viale Europa, 88100 Catanzaro, Italy; Paediatric Unit, Department of Experimental and Clinical Medicine, Magna Graecia University, Viale Europa, 88100 Catanzaro, Italy

**Keywords:** paediatric cardiology, multimodality imaging, myocarditis, advanced echocardiography, CMR

## Abstract

Paediatric myocarditis is a challenging and heterogeneous condition, with varied clinical presentations ranging from mild symptoms to life-threatening complications such as heart failure, arrhythmias, and sudden cardiac death. Effective management hinges on early diagnosis, appropriate treatment, and ongoing monitoring, which can be significantly enhanced through multimodal imaging techniques. This review emphasizes the crucial role of advanced imaging in the diagnosis, prognostication, and management of paediatric myocarditis. While traditional echocardiography remains the first-line imaging tool, it is often insufficient in detecting subtle myocardial changes and it does not allow the identification of myocardial inflammation and fibrosis, particularly in cases with preserved left ventricular function. Recent advancements, including speckle-tracking echocardiography, provide enhanced sensitivity for detecting early signs of myocardial dysfunction, even in the absence of overt abnormalities. Cardiovascular magnetic resonance (CMR) has emerged as a cornerstone in the non-invasive evaluation of myocarditis, offering unparalleled tissue characterization. Indeed, CMR provides critical insights into myocardial oedema, necrosis, and fibrosis, which are essential for confirming the diagnosis, stratifying prognosis, and guiding therapy. Parametric mapping techniques allow for highly accurate detection of myocardial fibrosis (native T1 mapping) and inflammation (T2 mapping), even in the absence of gadolinium contrast, making it particularly valuable in paediatric patients. In conclusion, multimodality imaging, integrating echocardiography and CMR, allows for a comprehensive understanding of disease severity, guiding treatment decisions and optimizing long-term outcomes. This review underscores the importance of a tailored, imaging-driven approach to managing paediatric myocarditis, ensuring the best possible care for this special population.

## Graphical Abstract

**Graphical Abstract:**
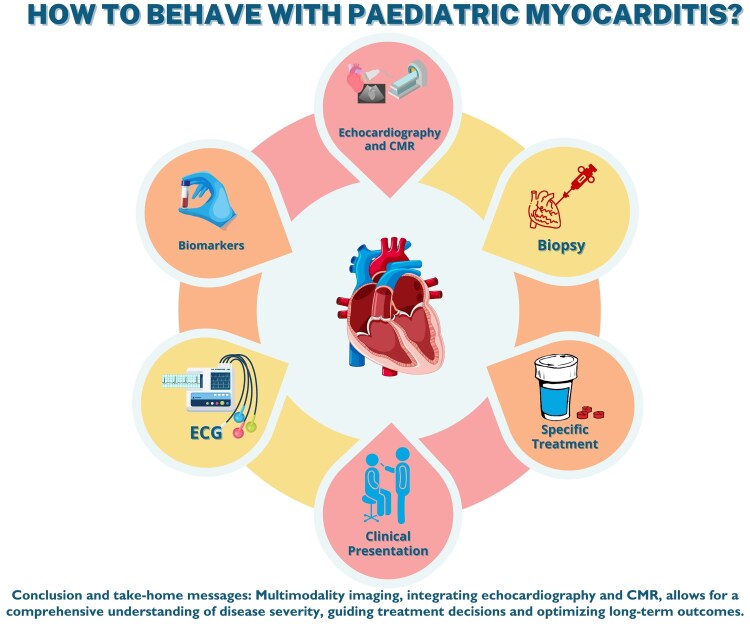
Understanding how to approach paediatric myocarditis involves both clinical considerations and imaging methods. Multimodality imaging, integrating a comprehensive physical examination, laboratory testing, cardiac biomarkers, ECG, and biopsy, provides a comprehensive understanding of disease severity, guiding treatment decisions and optimizing long-term outcomes.

## Introduction

The management of myocarditis in children poses several challenges for clinicians, ranging from establishing an initial diagnosis to selecting the most appropriate treatment and determining optimal follow-up care strategies. While some children may experience a complete and spontaneous recovery, others may have irreversible damage to the heart muscle. This comprehensive review seeks to offer clinicians practical guidance on the diagnosis and management of paediatric myocarditis, with a particular focus on imaging methods, and taking into account the unique aspects of the disease, including long-term repercussions on the patient’s overall health. By equipping physicians with this essential knowledge, we aim to empower them to deliver the most effective and appropriate care to their paediatric patients.

### Definitions, epidemiology, and pathophysiology

Myocarditis is an inflammatory disorder of the myocardium, defined by the presence of myocardial oedema alongside myocardial damage or necrosis.

The exact incidence of paediatric myocarditis remains unclear and is likely underestimated due to the absence of standardized diagnostic protocols and the possibility that milder cases may either remain asymptomatic or present with non-specific symptoms.

A global study on disease incidence in 2017 reported an incidence of 1.804 million cases and a prevalence of 3.071 million cases worldwide.^1^

A study conducted in Finland suggests that the incidence of myocarditis increases significantly after the age of seven, with a notable peak during early adolescence, particularly between the ages of 14 and 15.^2^

Several studies indicate that male children are more frequently affected by myocarditis across all age groups.

A recent study^3^ on myocarditis in children aged 0–14 years reveals a decline in myocarditis-related mortality between 1990 and 2021. However, the same study^3^ highlights a rise in the incidence reported for 2021, with a total of 155.45 cases per 1000 children worldwide and the importance of early detection and treatment of myocarditis in infants under one year of age, as this group exhibits significantly higher mortality rates compared with other age groups, despite their relatively low incidence.^3^

Patients of any age can be affected; however, most studies show a bimodal age distribution with peaks in neonatal and adolescence.^4–7^

Although the aetiology of myocarditis often remains undetermined, a wide range of infectious or non-infectious agents can cause the disease (*Table 1*).

**Table 1:** Causes of myocarditis^8^

Infectious causes	
Viral	Adenovirus, arbovirus, chikungunya virus, echovirus, enterovirus, hepatitis B, hepatitis C, herpes viruses, human immunodeficiency virus, influenza, mumps virus, parvovirus B19, poliovirus, rabies virus, respiratory syncytial virus, rubella virus, rubeola virus, varicella, variola virus, coronavirus
Bacterial	*Brucella*, *Burkholderia pseudomallei*, *Chlamydia*, *Clostridium*, *Corynebacterium diphtheria*, *Francisella tularensis*, Gonococcus, *Haemophilus influenzae*, *Legionella*, *Mycobacterium tuberculosis*, *Neisseria meningitidis*, *Salmonella*, *Staphylococcus*, *Streptococcus* A *Streptococcus pneumonia*, *Tetanus*, *Vibrio cholera*
Spirochetal	*Borrelia burgdorferi*, *Borrelia recurrentis*, *Leptospira*, *Treponema pallidum*
Fungal	*Actinomyces*, *Aspergillus*, *Blastomyces*, *Candida*, *Coccidioides*, *Cryptococcus*, *Histoplasma*, *Nocardia*, *Sporothrix schenckii*, *Strongyloides stercoralis*
Rickettsial	*Coxiella burnetiid*, *Rickettsia prowazekii*, *Rickettsia rickettsii*
Protozoal	*Balantidium*, *Entamoeba histolytica*, *Leishmania*, *Plasmodium falciparum*, Sarcocystis *Toxoplasmosis gondii*, *Trypanosoma brucei*, *Trypanosoma cruzi*
Non-infectious causes	
Autoimmune	Churg–Strauss syndrome, giant cell myocarditis, inflammatory bowel disease, insulin dependent diabetes mellitus, Kawasaki disease, lupus, myasthenia gravis, polyglandular autoimmune Syndrome, polymyositis, rheumatoid arthritis, sarcoidosis, scleroderma, thyrotoxicosis Wegener’s granulomatosis
Hypersensitivity drugs	Antibiotics (beta-lactams, tetracyclines, fluoroquinolones, vancomycin, macrolides, anti-tubercular agents, sulfonamides), central nervous system agents (benzodiazepines, carbamazepine, clozapine, methyldopa, phenytoin, tricyclic antidepressants), others (aminophylline, colchicine, dobutamine, lidocaine, phenylbutazone, thiazide diureties, immune checkpoint inhibitors)
Hypersensitivity drugs of abuse	Alcohol, cannabis, cocaine, hydrocarbon inhalants
Hypersensitivity-toxins	Arsenic, insect venom
Allergens	Tetanus toxoid, vaccines. serum sickness
Allo-antigens	Heart transplant rejection

Acute myocarditis usually recognizes an infectious origin. In particular, over time, adenovirus and enteroviruses have been replaced by parvovirus B19 and human herpesvirus 6 as the most frequent viral causes of paediatric myocarditis.^8^

The COVID-19 pandemic has highlighted SARS-CoV-2 as a significant cause of myocarditis, particularly in the context of multisystem inflammatory syndrome in children (MIS-C). The incidence is higher in children infected with COVID-19 compared with those not infected, though it remains relatively rare.^9^

Myocarditis can be caused by various non-infectious factors, including autoimmune disorders, hypersensitivity reactions, drugs, and toxins. In children with lupus erythematosus, ∼10.8% may experience myocarditis, pericarditis, or both.

One rare, autoimmune-mediated form of myocarditis is giant cell myocarditis, which, though uncommon in children, can be fatal if not promptly diagnosed and treated.

Hypersensitivity myocarditis, typically associated with drug use, is characterized by eosinophilic infiltration on biopsy. While drugs are the most common trigger, this condition can also be caused by toxins, infections, and even cancer, though these causes are less frequent.^10^

In *Table 2*, the most important key differential diagnoses of myocarditis in paediatric patients have been outlined, highlighting the clinical, echocardiographic, CMR, and electrocardiographic features of each condition, along with their differences from myocarditis.

**Table 2:** Differential diagnosis of paediatric myocarditis: a comparative table

Differential diagnosis	Clinical characteristics	Echocardiographic features	CMR features	ECG findings	Differences from myocarditis
Viral myocarditis	Recent viral illness, fever, dyspnoea, fatigue.	Global hypokinesis, variable chamber dilation, LV dysfunction.	Myocardial oedema, patchy LGE in non-ischaemic distribution.	Sinus tachycardia, ST-T changes, low voltage QRS.	N/A (reference condition).
Dilated cardiomyopathy (DCM)	Chronic onset, family history of cardiomyopathy, symptoms of heart failure.	Ventricular dilation with reduced systolic function.	LGE in non-inflammatory patterns.	Non-specific ST-T wave changes, arrhythmias.	Chronic presentation; no recent illness or inflammatory markers.
Hypertrophic cardiomyopathy (HCM)	Family history, asymmetric septal hypertrophy, often asymptomatic until adolescence.	Asymmetric hypertrophy, preserved systolic function, LVOT obstruction.	Absence of inflammation or oedema, myofiber disarray pattern.	Deep Q waves in lateral leads, LV hypertrophy.	No oedema or inflammation; septal hypertrophy is hallmark.
Pericarditis	Pleuritic chest pain, pericardial friction rub, fever.	Normal ventricular function, pericardial effusion.	Pericardial enhancement without myocardial involvement.	Diffuse ST elevation, PR segment depression.	Pericardial enhancement; no myocardial dysfunction.
Pompe disease (metabolic)	Progressive heart failure, cardiomegaly, muscle weakness, elevated CK, transaminases.	Cardiomegaly, left ventricular hypertrophy.	Left ventricular hypertrophy, no myocardial inflammation.	A short PR interval and tall and broad QRS complexes are seen on ECG and often considered diagnostic.	May present with poor feeding, muscle weakness, and hypertrophic cardiomyopathy-like features. Genetic testing is needed for diagnosis.
Sepsis-induced cardiomyopathy	Signs of systemic infection (fever, hypotension, elevated inflammatory markers).	Diffuse hypokinesis without significant ventricular dilation.	Minimal LGE, reversible myocardial dysfunction.	Sinus tachycardia, non-specific ST-T changes.	Related to systemic infection, rapid reversibility with sepsis resolution.
Congenital aortic stenosis	Heart murmur, left-sided heart failure symptoms, exercise intolerance.	Ventricular dilation with reduced systolic function. Left ventricular hypertrophy. Restricted aortic valve mobility, possible aortic regurgitation.	No evidence of inflammation; normal myocardium.	Left ventricular hypertrophy, left atrial enlargement.	Fixed structural defect at the valve, no myocardial inflammation or oedema.
Coarctation of the aorta	Hypertension in upper limbs, weak/absent femoral pulses, differential BP between limbs.	Ventricular dilation with reduced systolic function. Narrowing of the aortic isthmus, left ventricular hypertrophy, post-stenotic dilation.	Aortic structural narrowing is visible, no myocardial inflammation.	Left ventricular hypertrophy.	Localized aortic narrowing without systemic myocardial involvement.
ALCAPA (anomalous left coronary artery from the pulmonary artery)	Infantile presentation with failure to thrive, feeding difficulty, signs of heart failure. Later presentation may include angina, syncope.	Left ventricular dilation, severe mitral regurgitation, impaired LV function.	Coronary steal phenomenon, perfusion defects, ischaemic changes without inflammation.	Deep Q waves in anterolateral leads, ST depression or T-wave inversion.	Structural coronary abnormality with ischaemic findings; no inflammatory oedema or myocarditis-related patterns.

### Clinical presentation in the paediatric population

**Figure 1:**
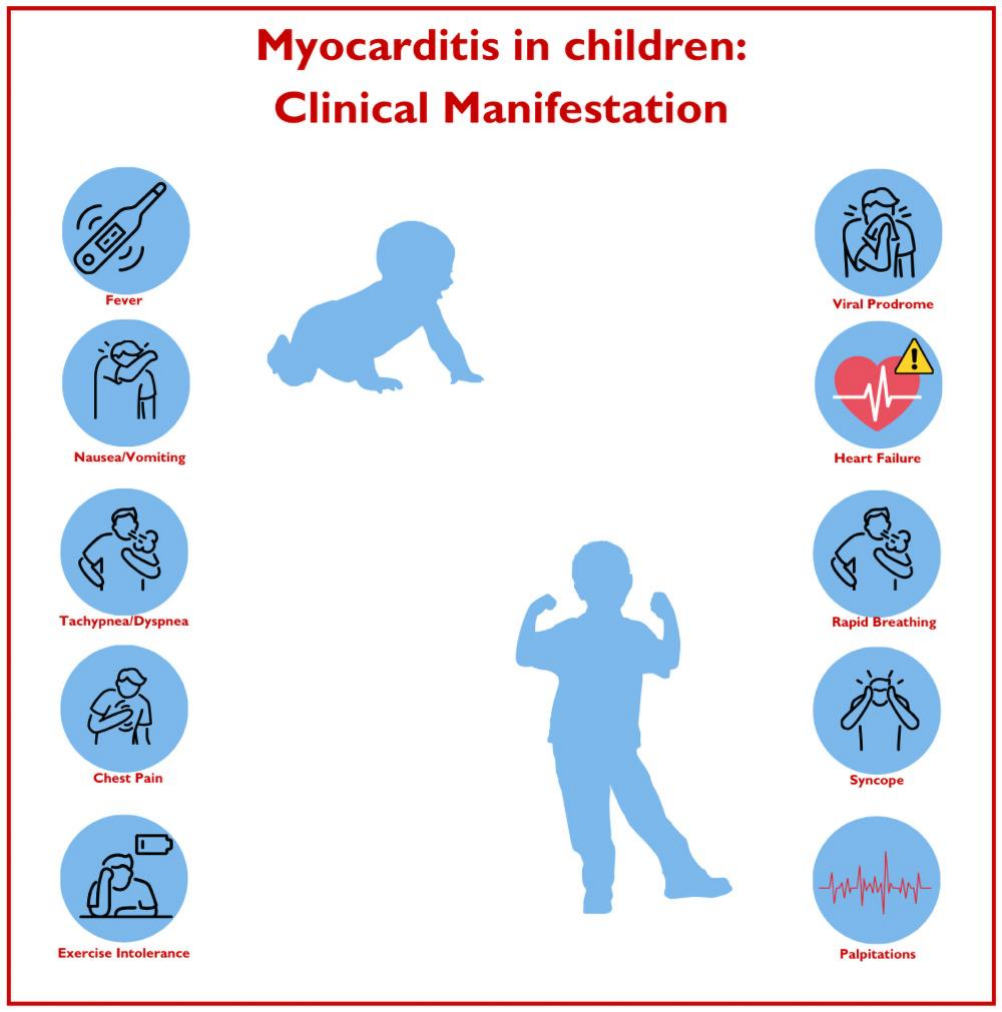
Clinical presentation of paediatric myocarditis. In the paediatric population, myocarditis shows two incidence peaks: the first during the neonatal period and the second in adolescence. Sudden cardiac death is most frequent in the first months of life. In neonates, common symptoms include heart failure, irritability, and poor feeding. In contrast, adolescents typically present with symptoms such as breathing difficulties, arrhythmias, and chest pain, with older children also experiencing exercise intolerance. Fever and viral prodromes frequently precede these symptoms across all age groups, providing an important diagnostic context.

The clinical presentation of myocarditis is extremely heterogeneous (*Figure 1*), ranging from asymptomatic manifestations to complex conditions such as sudden cardiac death and cardiogenic shock.^4^

However, especially in the paediatric setting, signs and symptoms can be non-specific and confusing. In particular, in adolescents, the typical presentation modality involves a recent story of respiratory or gastrointestinal illness with fever, myalgia, abdominal pain, vomiting, loss of appetite, malaise, cough, rhinorrhoea that precede by approximately two weeks, the onset of cardiac dysfunction which generally manifests itself with dyspnoea at rest, exercise intolerance, syncope, hepatomegaly, chest pain (especially if pericarditis coexists), tachypnoea, and persistent tachycardia.^11^

**Figure 2:**
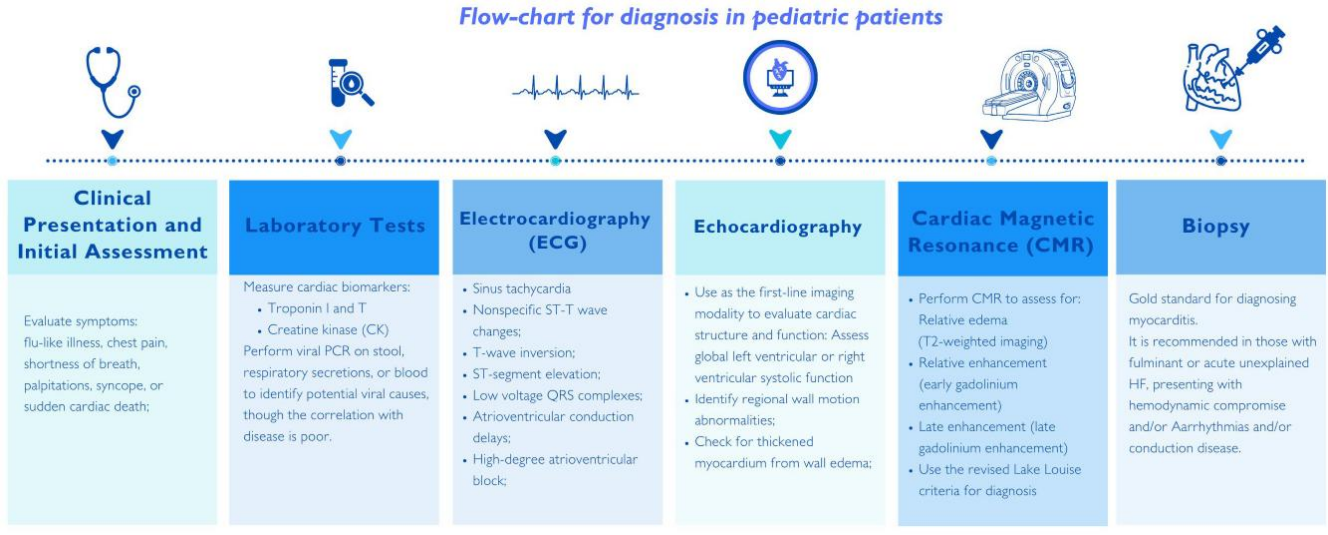
Flowchart for the diagnosis of myocarditis in children. The figure depicts a flowchart for the diagnosis of myocarditis in paediatric patients, along with a brief description of the key points for each step that are critical for making an accurate diagnosis.

Three distinct clinical manifestations can be distinguished:

– Acute myocarditis: characterized by heart failure features, electrocardiographic findings suggestive of cardiac damage, and previous viral infection.^12^

– Fulminant myocarditis: with acute onset and it is the result of a widespread myocardial inflammation; with hypotension, weak pulses, poor perfusion, and acidosis until a progressive cardiovascular collapse with severe haemodynamic compromise requiring inotropic or mechanical circulatory support.^8^

– Chronic myocarditis: defined by symptoms like chest pain and palpitations, and persistent cardiac inflammation histologically documented. We have included a flowchart (*Figure 2*) for the diagnosis of myocarditis in paediatric patients, along with a brief description of the key points for each step that are critical for making an accurate diagnosis.

### Electrocardiographic features

**Figure 3:**
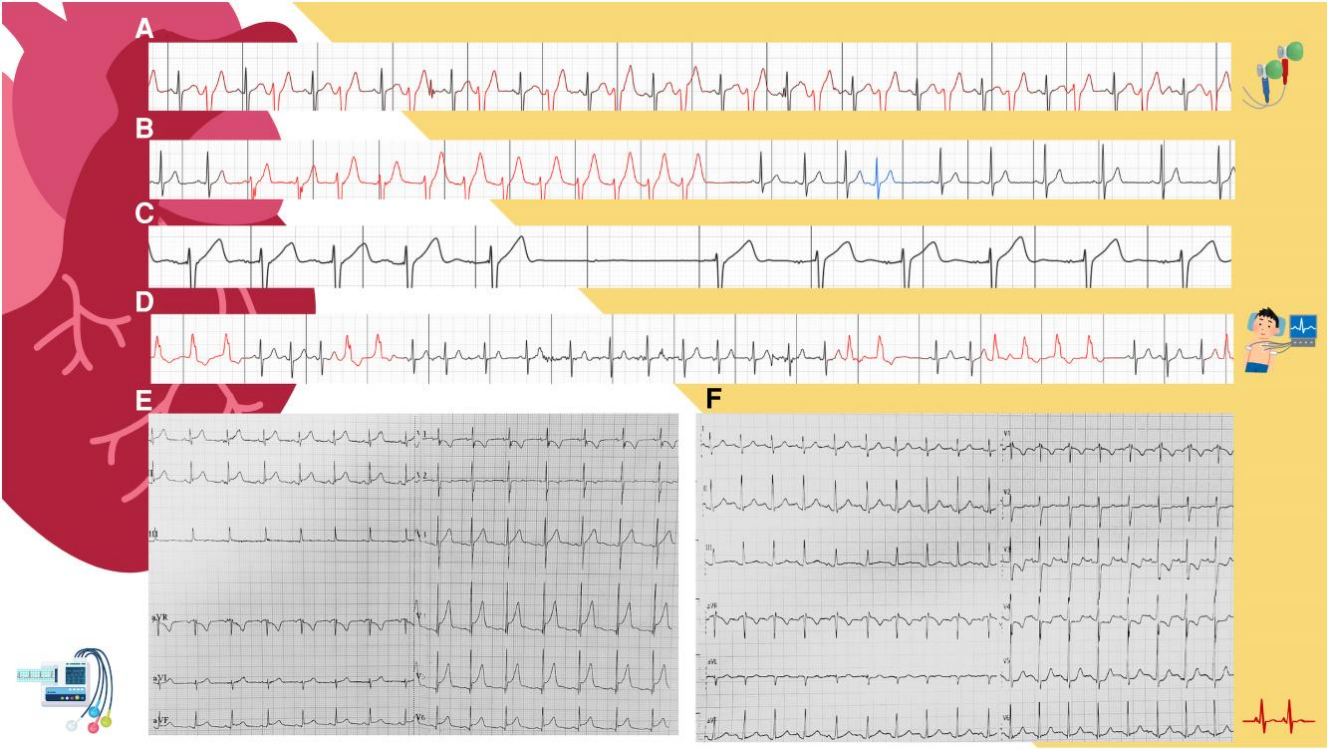
ECG features. Myocarditis can lead to both bradyarrhythmias and tachyarrhythmias. The figure demonstrates arrhythmia detection via Holter monitoring in a patient with acute myocarditis, showing the following patterns: (*A*) bigeminy, (*B*) supraventricular tachycardia, (*C*) atrioventricular (AV) block, and (*D*) frequent ventricular premature beats. Electrocardiograms performed upon admission (*E*) and 4 days later (*F*) in a 9-year-old patient reveal the resolution of ST elevation in lateral leads and the emergence of T-wave inversion in leads V2, V3–V4, and III, indicating evolving myocardial injury.

In children with myocarditis, ECG may show a variety of abnormalities (*Figure 3*), none of which are pathognomic.^12^

These may include:

– Repolarization abnormalities and extended QT intervals;

– Low voltage QRS complexes;

– Fractionated QRS;

– ST-T wave changes; and

– ST-segment elevation.

Tachyarrhythmias and atrioventricular (AV) blocks may occur in both acute and chronic phases with a variable severity from asymptomatic to life-threatening forms.

The presence of tachyarrhythmias is associated with poor prognosis, with increased in-hospital mortality.^13^ Both supraventricular and ventricular arrhythmias (VA) have been described, although the latter are prevalent in this scenario.

AV blocks represent another possible complication. In most cases they are transient, however, in some cases, the electric disturbances can be permanent and require pacemaker (PM) implantation. The average recovery time observed for both VA and AV block during the acute phase is 5–6 days.^14^

### Cardiac biomarkers

Cardiac-specific troponins T and I and markers of inflammation (C-reactive protein or erythrocyte sedimentation rate and white blood cell count) are frequently increased. However, no specific biomarker for myocarditis is currently available, and elevated values of troponins cannot differentiate myocarditis from other causes of acute myocardial dysfunction, injury, or ischaemia. Finally, normal troponins are not able to rule out the presence of myocarditis. Additionally, heart-related peptides like the N-terminal fragment of B-type natriuretic peptide might be higher in myocarditis, aiding in differentiating between heart-related and other causes of respiratory symptoms in children.^8,12^

### Echocardiographic imaging: the key to early detection and better outcomes

Transthoracic echocardiography is the first-choice imaging modality in paediatric myocarditis. In general, there are no specific echocardiographic features of myocarditis, and many patients with milder forms of the disease may exhibit normal echocardiographic findings.

Pinamonti *et al*.^15^ described forms of myocarditis in adult patients presenting with echocardiographic patterns of dilated, hypertrophic, and restrictive cardiomyopathy. Several studies in paediatric patients indicate that impaired ventricular function, involving either the left or right ventricle, is associated with adverse outcomes, including an increased risk of mortality and the need for transplantation.^16^

In patients with preserved left ventricular ejection fraction (LVEF), additional echocardiographic parameters that warrant evaluation during the course of myocarditis include diastolic dysfunction, functional valvular regurgitation, myocardial wall thickness, and the dimensions of both atrial and ventricular chambers. Moreover, with echocardiography, we can exclude pericardial involvement such as pericardial effusion and/or pericardial frank hyper-echogenicity. Of note, previous studies have shown that cardiac enlargement is associated with adverse cardiovascular events.

Felker *et al*.,^17^ suggest that the evaluation of left ventricular dimensions can be useful as a method to differentiate between two distinct manifestations of myocarditis:

– Fulminant myocarditis: characterized by the presence of a non-dilated left ventricle that is thickened and hypo-contractile, resulting from an intense inflammatory response that leads to interstitial oedema and a loss of ventricular contractility;

– Acute myocarditis: characterized by marked left ventricular dilatation and normal wall thickness.

Moreover, the role of echocardiography in the setting of myocarditis is crucial before proceeding to any biopsy to exclude pericardial effusion and intracavitary thrombi.

At last, it plays a key role in the management and follow-up of myocarditis patients. Indeed, echocardiographic re-evaluation during follow-up is the cornerstone of optimizing medical management and improving outcomes.

### Advanced echocardiography: enhancing diagnosis and management of paediatric myocarditis

Recently, speckle-tracking echocardiography has been introduced into clinical routine. It has been widely demonstrated this technology offers increased accuracy in diagnosing left and right ventricular systolic and diastolic dysfunction compared with conventional 2D echocardiography, across various cardiovascular diseases.

Several studies have emphasized the importance of routinely using new measures of cardiac deformation as indicators of subclinical left ventricular dysfunction. These measures have been validated, particularly in adolescents and young adults with acute myocarditis diagnosed by cardiac magnetic resonance (CMR) and preserved ejection fraction (EF), a condition that may often escape timely diagnosis.^18^

According to Løgstrup *et al*.^19^ in a population of 28 young adults suffering from acute myocarditis, the presence of oedema assessed by CMR correlates significantly with the global longitudinal strain (GLS) (r = 0.65; *P* < 0.001).

**Figure 4:**
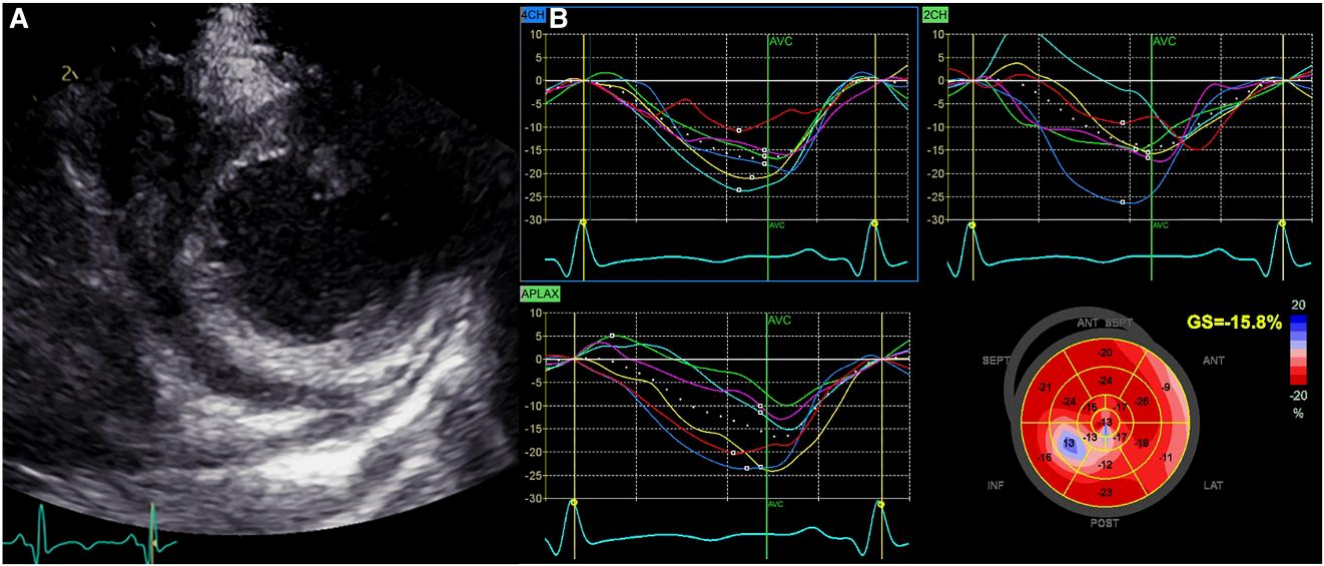
Standard echocardiography and speckle-tracking analysis in paediatric myocarditis. (*A*) Parasternal short-axis view of the left ventricle on 2D echocardiography. 2D parasternal short-axis view of the left ventricle showing segmental hyper-echogenicity in the inferior wall. (*B*) Speckle-tracking assessment of left ventricular global longitudinal strain (GLS) in a child with myocarditis. Speckle-tracking analysis with a bull’s-eye plot for the same patient reveals a reduced global longitudinal strain (−15.8%) and pronounced segmental abnormalities in the inferior segments of the left ventricle.

Similar findings were observed in a population of children and adolescents with focal myocarditis and normal ejection fraction, where a significant reduction in global and regional myocardial deformation was identified notwithstanding normal traditional parameters of left ventricular function (EF 61 ± 5%).^20^ Of note, the majority of patients exhibit significantly reduced regional longitudinal strain in the infero-septal, and infero-lateral walls (GLS = −16.2 ± 5.5%) as compared with the mean of the other myocardial regions (− 23.5 ± 4.2%; *P* < 0.05).^20^ These findings underscore the value of assessing regional and global left ventricular myocardial deformation using longitudinal strain in young patients. Importantly, speckle-tracking analysis offers enhanced insights into the location and extent of cardiac involvement in focal myocarditis compared with conventional echocardiography. Specifically, longitudinal strain analysis conducted at the time of admission provides robust information on the localization and severity of myocardial oedema in paediatric patients with acute myocarditis, and proves particularly valuable in the differential diagnosis of chest pain, especially in the presence of bio-humoral and electrocardiographic abnormalities. In paediatric and young adult populations, these abnormalities (especially in infero-postero-lateral regions) are rarely associated with coronary artery disease but can effectively guide the diagnosis of myocarditis (*Figure 4*).

Unlike GLS, there is limited data regarding left atrial strain imaging among children with myocarditis. Several studies on the paediatric population have attempted to analyse diastolic function by studying the left atrial chamber and its function.^21^ A recent comparison study conducted by Meindl *et al*.^18^ highlighted that in patients with acute myocarditis, left atrial reservoir function, left atrial conduit function, and left atrial stiffness index as well as left atrial filling index were impaired compared with healthy controls, indicating the presence of diastolic dysfunction.

Finally, 3D echocardiography can provide a more accurate assessment of ventricular volumes and LVEF, compared with 2D evaluation, although it tends to slightly underestimate volumes compared with CMR.^22^

The integration of advanced echocardiographic evaluation, given the heterogeneous modalities of presentation of the disease, could improve the diagnostic accuracy in patients suffering from acute myocarditis, when conventional echocardiographic parameters of the left ventricle do not show any alteration and in cases where the use of CMR is limited.

### Challenges of advanced echocardiography in paediatric age

Advanced echocardiography in paediatrics poses challenges due to variations in anatomy, heart rate, patient cooperation, and, if coexisting congenital heart disease, and abnormal ventricular geometry often necessitating specialized techniques or sedation. In particular, higher heart rates in paediatric patients necessitate increased frame rates during image acquisition to ensure the accuracy and reproducibility of strain measurements. Specific paediatric-specific protocols and normative data may complicate interpretation, while specific advanced modalities like 3D imaging and speckle-tracking require expertise and are not universally accessible.

### Cardiovascular magnetic resonance: the non-invasive revolution in paediatric myocarditis diagnosis

Cardiovascular Magnetic Resonance has emerged as a valuable, non-invasive diagnostic alternative to endomyocardial biopsy, yielding good sensitivity and specificity.

**Figure 5:**
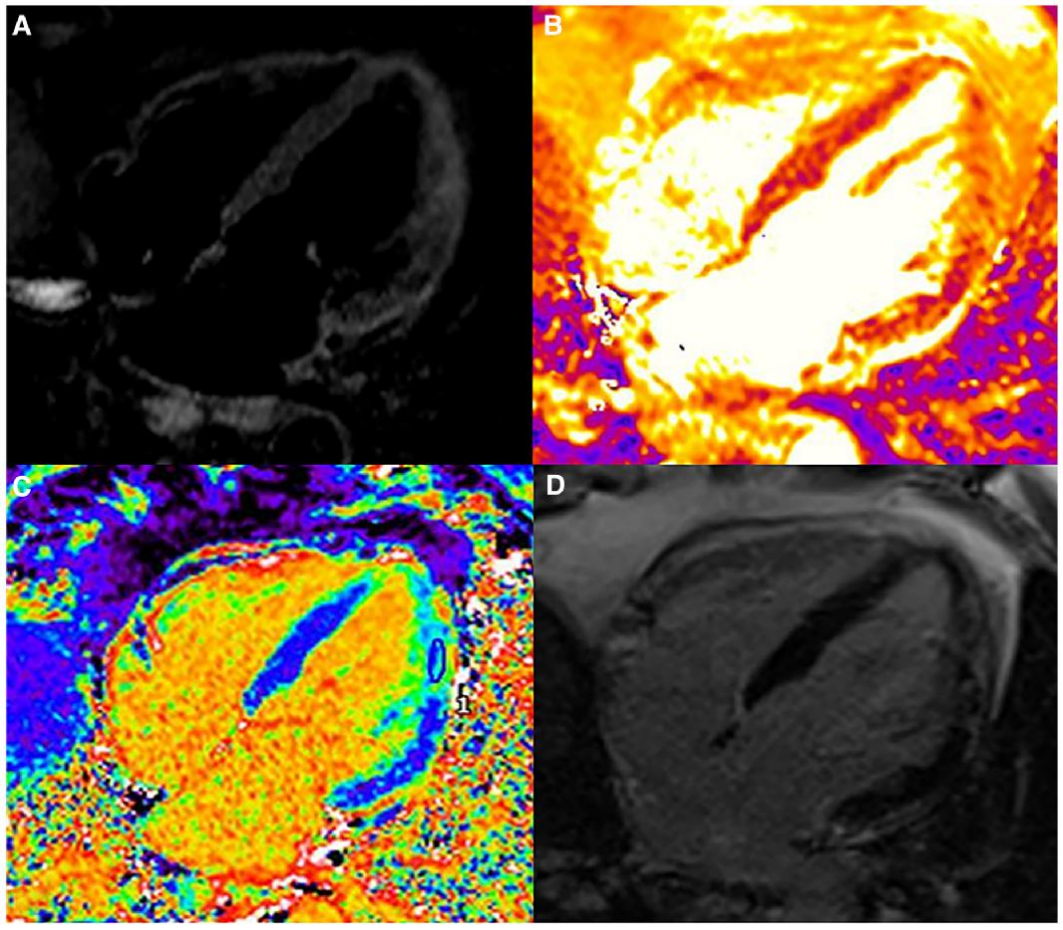
Cardiovascular MRI findings in myocarditis. Cardiovascular MRI in a patient with myocarditis showing high signal intensity on T2-weighted images in the anterolateral wall (STIR-T2, *A*) and increased T2 mapping values (70 ms, *B*) and ECV values (40%, *C*) in the same area. After injection of gadolinium contrast, enhancement with a non-ischaemic pattern involving the anterolateral wall is highlighted (*D*).

**Figure 6:**
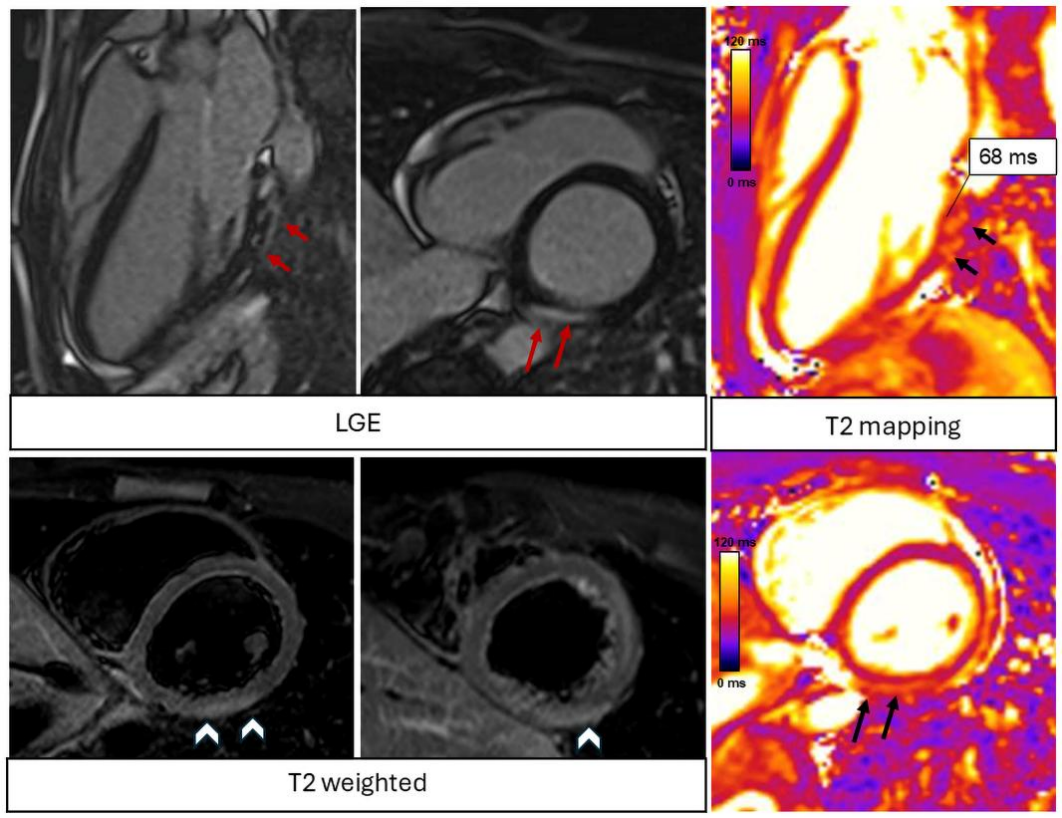
Cardiac magnetic resonance images of a patient with acute myocarditis. At basal infero-lateral wall, there is evidence of increased signal intensity with a subepicardial pattern in both T2-weighted and LGE images in keeping with the diagnosis of acute myocarditis. Increased T2 mapping values in the same areas confirm the presence of myocardial oedema. LGE, late gadolinium enhancement.

In addition to the quantification of biventricular volumes and function, CMR has the unique advantage of providing myocardial tissue characterization, pointing out inflammatory changes such as myocardial oedema, hyperaemia, and cardiomyocyte necrosis. As a result, the diagnostic paradigm based on the ‘Dallas criteria’, is currently shifting toward a multi-parametric and multimodal approach, where CMR is the key imaging modality to capture the presence, evolution, and distribution of myocardial inflammation (*Figures 5* and *6*). This may be particularly relevant for the paediatric population, given the absence of ionizing radiation.^23^

### Cardiovascular magnetic resonance diagnostic criteria

According to the revised Lake Louise Criteria, the presence of one T2-based criterion associated with at least one T1-based criterion allows the diagnosis of acute myocarditis. Even if only one (either T2- or T1-based) criterion is satisfied, the diagnosis could still be made in the context of a highly suspicious clinical scenario, although with less specificity.^23,24^ In detail, the T2 criteria include: (i) regional high T2 signal intensity; (ii) global T2 signal intensity ratio ≥ 2.0 in T2-weighted images, and (iii) regional or global increase of myocardial T2-relaxation time; the T1 criteria include: (i) regional or global increase of native myocardial T1 relaxation time or extracellular volume (ECV); or (ii) areas with high signal intensity in a non-ischaemic distribution pattern in late gadolinium enhancement (LGE) images.^24^

Evidence of concomitant pericarditis is considered supportive criteria. This can be identified as pericardial enhancement on both T2-weighted and LGE sequences (acute process), or isolated pericardial LGE (subacute/chronic process). the former suggesting acute pericarditis, the latter subacute/chronic pericarditis. Pericardial effusion can be present but is not mandatory for the diagnosis.^24^

Among the novelties of the revised Lake Louise Criteria is the inclusion of parametric mapping sequences (T1, T2 mapping, and ECV), which yield a higher diagnostic accuracy when combined with the original criteria.^23,24^ Native T1 mapping has also the unique advantage to allow the assessment of myocardial fibrosis without the use of gadolinium; This non-contrast approach has demonstrated to have a good diagnostic accuracy, not significantly different from the LGE-based one. Of note, the Lake Louise Criteria have been originally validated in adult patients, raising concerns about their actual applicability in the paediatric setting. In this regard, a study by Cornicelli *et al*.,^25^ confirmed the feasibility and accuracy of both T1 and T2 parametric mapping in children, with significantly elevated mean global values of both parameters in patients with clinically suspected acute myocarditis compared with healthy controls. Similarly to what was already demonstrated in adults, the use of CMR parametric mapping techniques measurably increased CMR diagnostic yield when compared with original Lake Louise Criteria, thus strengthening the use of these novel techniques also in children.^25^

### CMR in risk stratification and follow-up

**Figure 7:**
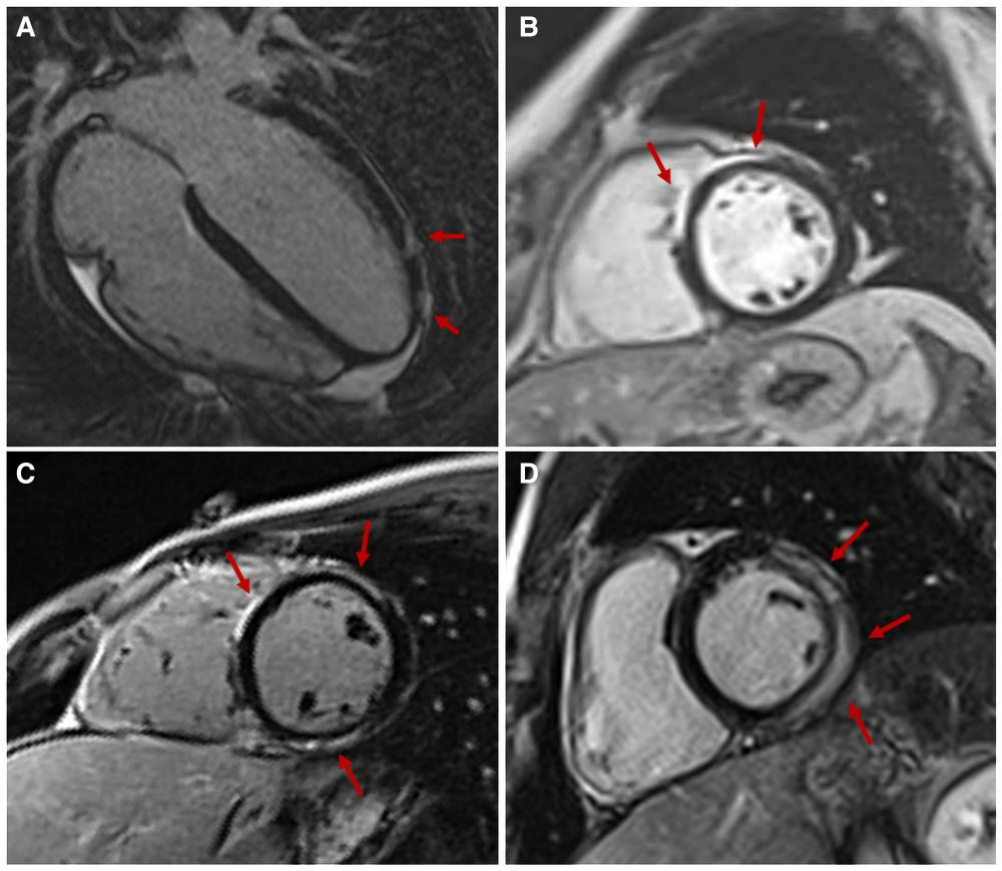
LGE patterns in myocarditis. Patients with myocarditis presenting LGE with patchy distribution in the apical lateral wall (*A*), subepicardial LGE in the anteroseptum (*B*), multiple areas of LGE involving both the RV septal wall, the anterior and inferior LV wall (*C*-*D*), and mid-wall LGE in the LV lateral wall with a ring-like pattern. LGE, late gadolinium enhancement; LV, left ventricular; RV, right ventricular.

CMR can also provide some important prognostic information. LGE is the most extensively studied prognostic marker in patients with myocarditis^26^ (*Figure 7*). Its distribution typically follows a non-ischaemic pattern with a subepicardial, mid-wall, or patchy distribution, with localization and extent that have been correlated to a higher risk of early and late complications. In particular, a mid-wall distribution and anteroseptal location of the LGE have been associated with a higher risk of VA, death, and failure of recovery compared with the infero-lateral localization or patchy distribution, also among patients with preserved left ventricular ejection fraction on admission.^27^ During follow-up CMR imaging can assess recovery of LV function, resolution of myocardial inflammation, and presence and extension of myocardial scarring. This is important since areas of increased signal intensity in LGE sequences during the acute event may be secondary to myocardial oedema and not necessarily correspond to myocardial fibrosis. In a study enrolling 68 children with acute myocarditis a follow-up CMR demonstrated in fact complete resolution of tissue abnormalities in about one in four patients. Increased T2-relaxation time and presence of LGE (particularly with a mid-wall pattern) were more frequently observed in case of absent recovery^28^ Ongoing inflammation and persistent fibrosis at LGE can however be observed even after normalization of other routinely performed tests including LVEF, highlighting the incremental role of CMR serial evaluation over echocardiographic examination.^29^ A ring-like LGE pattern, defined as at least 3 contiguous positive LGE segments in the same short-axis slice, is often seen in genetic cardiomyopathies, including desmoplakin-related cases. Its recognition is therefore crucial and should prompt further evaluation, such as genetic testing.^30^

### Challenges of using CMR in paediatrics

Despite the advantages of a non-invasive approach, CMR has some limitations. Firstly, it cannot provide an aetiological diagnosis, which is crucial for determining the optimal therapeutic strategy, particularly in cases of fulminant myocarditis.

Furthermore, it’s necessary to consider limits related to the methodology itself.

The size of young children can pose difficulties in optimizing CMR sequence parameters and can significantly affect the spatial resolution of the image, due to a reduction of the signal-to-noise ratio or higher heart rates which can pose several difficulties for temporal resolution and for obtaining still images during mid-diastole, avoiding cardiac motion artefacts.

Young children can present difficulties in holding their breath, this contributing again to generate cardiac motion artefacts.^23^ Lastly, children under 6 years of age undergoing CMR routinely require sedation or general anaesthesia, which carries low but potentially life-threatening risks.

### Endomyocardial biopsy in paediatric myocarditis: controversies, challenges, and advancements

In the modern era, endomyocardial biopsy (EMB) remains the gold standard for diagnosing myocarditis,^8^ although its use in children remains controversial.^31^

In children EMB is recommended in those with fulminant or acute unexplained HF, presenting with haemodynamic compromise and/or arrhythmias and/or conduction disease.

The timing of EMB is debated, but its diagnostic yield is higher if performed within 2 weeks of symptom onset, preferably after haemodynamic stabilization.

Previously, Dallas histopathologic criteria were used, however, a 2013 European Society of Cardiology (ESC) Position Paper has outlined the importance of the characterization and quantification of cardiac inflammation using immunohistochemistry and viral genome analysis with quantitative PCR, both for the diagnosis and the use of specific treatments.

ESC experts recommended adopting the Marburg criteria (>14 mononuclear leukocytes/mm^2^ with the presence of >7 T lymphocytes/mm^2^ on bioptic samples) to enhance EMB sensitivity.^32^

CMR imaging study has shown that myocarditis primarily affects the free wall of the left ventricle, an area that is inaccessible using standard endomyocardial biopsy. Additionally, left ventricular EMB is rarely performed in children.

The use of CMR-guided EMB enhances the diagnostic yield by targeting specific areas of myocardial involvement, especially in cases of focal myocarditis.^33^

The right internal jugular vein is the most common percutaneous access site for the right ventricular EMB. Left ventricular EMB is rarely performed in children.

The incidence of EMB complications in children ranges from 9 to 15.5%, with higher percentages in infants (up to 31.2%).^34^ Given the high incidence of serious complications, especially in small children, a careful evaluation of the risk-benefit ratio is essential.

### Treatment of acute myocarditis in paediatric population

Management of acute myocarditis in the paediatric population remains challenging due to a lack of clear evidence to significantly improve prognosis. The optimal treatment approach largely depends on the clinical presentation (*Figure 2*). Multimodality imaging plays a critical role in guiding treatment decisions, offering detailed and comprehensive insights into cardiac structure, function, and tissue characterization. This approach helps clinicians tailor therapy to the patient’s specific needs, ensuring a more precise and informed management strategy. If there are persistent abnormalities in ventricular function, inflammatory markers, or viral activity detected by PCR blood tests, further assessment through biopsy or CMR might be warranted. Conversely, if diagnostic tests normalize, it may be appropriate to discontinue medications aimed at reverse cardiac remodelling.^12,14^ CMR is particularly useful for detecting subtle damage and fibrosis that echocardiography might miss, impacting medication decisions. The optimal duration for medication after normalization of test results remains uncertain, given the potential for late pathological remodelling of the heart muscle.

For patients presenting with symptomatic heart failure, management typically includes a combination of ACEi, β-blockers, and aldosterone antagonists,^35^ see Supplementary data online, *Table S1* for posology. Among β-blockers, carvedilol shows a protective effect in these conditions. Diuretics could be helpful in congestive forms reducing preload. Sacubitril/valsartan, an angiotensin receptor-neprilysin inhibitor (ARNI), is a recognized therapy for heart failure with reduced left ventricular ejection fraction, especially after the acute phase of myocarditis. The PANORAMA-HF trial, the largest randomized, double-blind clinical study of paediatric heart failure (HF) to date, found no significant differences in the primary global rank endpoint between sacubitril/valsartan and enalapril for treating HF with systemic left ventricular systolic dysfunction in children.^36^ However, sacubitril/valsartan showed potential quality-of-life benefits and demonstrated an acceptable safety profile comparable to enalapril. Over 52 weeks, sacubitril/valsartan exhibited a favourable benefit-risk ratio in paediatric patients aged 1 to <18 years. Similarly, gliflozins (SGLT2 inhibitors), initially developed for diabetes, are gaining attention for their cardioprotective effects, including improved ventricular function and reduction of myocardial stress, making them a promising adjunctive therapy. While evidence in paediatric myocarditis remains limited, these therapies are being increasingly explored for their potential to address myocardial dysfunction in children.

On the other hand, in the setting of paediatric myopericarditis, the pericarditis treatment typically includes nonsteroidal anti-inflammatory drugs (NSAIDs) and colchicine, with careful monitoring for signs of myocardial involvement requiring specific management.

In severe cases presented as cardiogenic shock, continuous monitoring in the intensive care unit (ICU) is necessary, due to the potential worsening of clinical conditions, often associated with poor outcomes. Anticoagulation therapy may be recommended for severely decreased ejection fraction or atrial arrhythmias, while inotropic agents, typically milrinone, may be necessary for hypotension.

Inotropes with vasopressor properties, such as epinephrine and dopamine, are generally reserved for cases with refractory hypotension and cardiogenic shock, giving them more chronotropic and arrhythmogenic potential.

Data about specific immunotherapy treatment in children are lacking and inconclusive to allow evidence-based recommendations.^37^

However, intravenous immunoglobulin (IVIG) therapy is widely used, especially in severe myocarditis cases associated with considerable risk of mortality and morbidity. In multicentre observational studies, ∼70% of paediatric patients with myocarditis were treated with IVIG, and 20–30% received glucocorticoids.^38^ A meta-analysis including more than 1400 paediatric patients showed that IVIG therapy was associated with better survival.^39^

The use of glucocorticoids for acute myocarditis treatment is generally restricted to patients who don’t respond to IVIG or if other systemic autoimmune or inflammatory conditions are associated.

Paediatric studies on other examples of immunosuppressive regimens are limited. In one trial, patients received either standard treatment or were given one of three immunosuppressive therapies, prednisolone, prednisolone and azathioprine, or prednisolone and cyclosporin A.

Groups receiving immunosuppressive treatment with a second agent, in addition to prednisolone, showed significant improvement in haemodynamic parameters, as well as histological reduction of inflammation.

However, immunosuppression did not show a beneficial effect on the primary endpoint of this study (changing in LVEF at 28 weeks) and did not improve survival.

Antiviral therapies specific to myocarditis have not undergone rigorous clinical trials. However, considering their established efficacy in managing non-cardiac infections, it is reasonable to use these agents in cases of myocarditis when an active infection is identified, even in the absence of direct evidence of myocardial infection. In the end, it is mandatory to mention myocarditis associated with SARS-CoV-2 infection, whose management suggests treatment with both glucocorticoids, typically given first, and IVIG. It is demonstrated that the use of IVIG in MIS-C can improve cardiac outcomes in 70–95% of cases. Biologic therapies, such as IL-1 inhibitors, IL-6 inhibitors, and TNF-inhibitors, are considered as alternative options in patients who cannot receive glucocorticoids or have refractory diseases with persistent myocardial dysfunction.

### Tachyarrhythmias and AV blocks

Acute management of tachyarrhythmias depends on clinical presentation, with synchronized cardioversion/defibrillation recommended in haemodynamically unstable patients, regardless of the QRS complex width. According to the last European Paediatric Advanced Life Support guidelines^40^ during synchronized cardioversion is recommended to deliver 1 J/Kg body weight, double the energy for each subsequent attempt up to a maximum of 4 J/kg, while for defibrillation standard energy dose for shocks is 4 J/Kg considering escalating doses-stepwise increasing up to 8 J/Kg and max. 360 J—for refractory VF/pVT (i.e. more than five shocks needed). If the tachyarrhythmia is well tolerated further tests can be performed and proceeding either with synchronized cardioversion according to individual risk of anaesthesia/sedation or attempt a pharmacological therapy. Intravenous amiodarone, procainamide, or lidocaine has been used in paediatric population^40^; the dosage of the aforementioned drugs is reported in Supplementary data online, *Table S2*.

In addition, adequate supportive therapy should be provided, eliminating all possible predisposing or precipitating factors, such as hypoxia, hypovolemia, fever, and electrolyte imbalances. In patients that develop electrical storm treatment with beta-blockers, preferably non-selective beta-blockers like propranolol, combined with amiodarone may be useful. Landiolol, an ultra-short-acting β1-selective blocker, was recently found to be effective for arrhythmia suppression in patients with recurrent haemodynamically not-tolerated VTs resistant to amiodarone.

In patients developing AV block, temporary transvenous pacing should be considered, given the risk of haemodynamic compromise and ventricular dysfunction.^41^

### Mechanical circulatory support and heart transplant

Acute paediatric myocarditis is often associated with severe heart failure, requiring intensive care admission, inotropic support, and mechanical circulatory support.^12^

According to the patient’s clinical presentation, different strategies can be implemented. In case of cardiogenic shock, cardiorespiratory failure and sustained arrhythmias, the use of extracorporeal membrane oxygenation (ECMO) represents a valid option, as it can be rapidly deployed and the likelihood of a swift cardiac recovery in myocarditis is significantly greater when compared with cardiomyopathy. If the patient presents with low cardiac output syndrome and preserved pulmonary function, alternative options such as Impella (in babies > 30 kg) can also be considered.

Hospital discharge rates of nearly 80% have been reported in different studies on paediatric myocarditis requiring ECMO, with almost 60% of patients experiencing myocardial recovery.^42^ However, it should be noticed that survival in patients requiring > 2 weeks of ECMO support drops to <50% and factors such as arrhythmias, the need for dialysis, and advanced stages of organ perfusion have been associated with death. If weaning from ECMO is not possible, more durable devices such as Berlin Heart, HeartMate, or HeartWare HVAD should be taken into account. The choice of devices varies according to the patient’s weight, considering that the minimum required weight for device implantation is 2.5 kg, and clinical presentation. Indeed, biventricular support will be needed in case of severe biventricular failure; otherwise, the first approach consists of the implantation of left ventricular assist devices (VAD) and a right VAD is implanted afterward in case of refractory RV failure.

Even though recovery of myocardial function in paediatric myocarditis is highly likely, and scientific data have shown that weaning from long-term VAD is also possible in children with acute myocarditis, VADs are most often implanted as a bridge to heart transplant (HT) in this population.^43^ Of note, post-transplant survival has been reported as significantly worse in infants who received an HT while on ECMO compared with those on no support, while survival of patients on VAD support seems to be like those on no support.^44^ As for HT, despite the significant advancements in this field, this surgical option is still limited by the shortage of donors. Limited and conflicting data exist about outcomes for children with myocarditis listed for HT. A previous study reported that children with myocarditis who are listed for HT face an elevated risk of mortality while on the waitlist and after the HT compared with children with idiopathic dilated cardiomyopathy, with a reported survival of 83% at 1 year and 65% at 3 years post-transplant. Survival after HT also varies, and medial survival decreases from 22.3 years in patients who undergo HT < 1 year of age to 13.1 years for those aged >11 years. Thus, the decision to list a paediatric patient should be taken after a careful evaluation of medical and psychosocial aspects by a multidisciplinary team involving clinicians, surgeons, and psychologists.

## Conclusions

Myocarditis in children remains a difficult condition to diagnose and manage. Whether traditionally the diagnosis of myocarditis was based on histological evidence of immune cell infiltration, which resulted in myocyte damage and cell death, multimodality imaging holds the transformative potential to revolutionize the diagnosis of paediatric myocarditis, enabling earlier detection, more precise characterization, and tailored management strategies that could dramatically improve outcomes in children and young patients.

## Supplementary Material

qyaf025_Supplementary_Data

## Supplementary data

**Consent:** A written consent for publication of images and associated text has been obtained.

## Lead author biography

Dr. Rosalba De Sarro, MD, graduated cum laude from the University of Messina. She is currently a PhD student in Digital Technologies applied to Medicine at the Magna Graecia University of Catanzaro. A cardiovascular physician, she is engaged in research focused on echocardiographic cardiovascular imaging, particularly in the field of pediatric cardiology and congenital heart disease.

**Data availability:** The data underlying this article will be shared on reasonable request to the corresponding author.
