# Supplementary material for: How to behave with paediatric myocarditis: imaging methods and clinical considerations
Source: Eur Heart J Imaging Methods Pract. 2025 Apr 4;3(1):qyaf025. doi: 10.1093/ehjimp/qyaf025 (PMC11969066; doi:10.1093/ehjimp/qyaf025)
Supplement: qyaf025_Supplementary_Data [file qyaf025_supplementary_data.docx]

**Supplemental Material**

**Supplemental Table 1.** *Dosage of drugs in paediatric population*

| **Drug** | **Routes of administration** | **Posology** |
| --- | --- | --- |
| Furosemide | oral | 1-2 mg/kg every 6-12h |
|  | i.v.bolus | 0.5-2 mg/kg every 6-12h |
|  | i.v. continuous infusion | 0.1-0.4 mg/kg/h |
| Captopril | Oral | 0.3-2 mg/kg every 8h |
| Enalapril | Oral | 0.05-0.25 mg/kg every 12h |
| Carvedilol | Oral | 0.05 mg/kg every 12h |
| Metoprolol | Oral | 0.25 mg/kg every 12h |
| Spironolactone | Oral | 0.5-1.5 mg/kg every 12h |
| Nitroglycerin | i.v. continuous infusion | 0.5-10 ug/kg/min  0.5-4 pg/kg/min |
| Nitroprusside | iv. continuous infusion | 0.5-4 pg/kg/min |
| Hydralazine | i.v. bolus | 0.1-0.2 mg/kg every 4-6 h |
| Dobutamine | i.v. continuous infusion | 2.5-10 Mg/kg/min |
| Epinephrine | i.v. continuous infusion | 0.5-1 pg/kg/min  0.5-1 pg/kg/min |
| Milrinone | i.v. continuous infusion i.v. singol infusion Oral i.v. or subcutaneous i.v. singol infusion | 0.5-1 pg/kg/min |
| Immunoglobulin e.v | i.v. singol infusion | 2 gr/kg over 8 to 24h |
| Prednisolone | Oral | 2-2.5  mg/kg/24h then tapered off |
| Anakinra Infliximab | i.v. or subcutaneous | 5-10 mg/kg daily for three days |
| Infliximab | i.v. singol infusion | 5-10 mg/kg |

**Supplemental Table 2: Antiarrhythmic Drugs and Posologies**

| **DRUG** | **POSOLOGY** |
| --- | --- |
| Amiodarone | 5 mg/kg over 30 to 60 minutes followed by 5 to 15 g/kg/minute |
| Procainamide | 7 to 15 mg/kg over 1 hour followed by an infusion of 20 to 100 g/kg/minute |
| Lidocaine | 1 mg/kg followed by 20 to 50 ug/kg/minute |
